# Assessing the migrant mortality advantage among foreign-born and interprovincial migrants in Manitoba, Canada

**DOI:** 10.17269/s41997-022-00727-4

**Published:** 2022-12-27

**Authors:** Shantanu Debbarman, Heather Prior, Randy Walld, Marcelo L. Urquia

**Affiliations:** 1 Manitoba Centre for Health Policy, Community Health Sciences, Max Rady College of Medicine, Rady Faculty of Health Sciences, University of Manitoba, 408-727 McDermot Ave, Winnipeg, MB R3E 3P5 Canada; 2 Department of Economics, Faculty of Arts, University of Manitoba, Winnipeg, MB Canada; 3 Dalla Lana School of Public Health, University of Toronto, Toronto, ON Canada

**Keywords:** Mortality, Premature mortality, Immigration, Manitoba, Canada, Mortalité, mortalité prématurée, immigration, Manitoba, Canada

## Abstract

**Objectives:**

Studies on mortality differentials between international immigrants and non-immigrants produced mixed results. The mortality of interprovincial migrants has been less studied. Our objectives were to compare mortality risk between international immigrants, interprovincial migrants, and long-term residents of the province of Manitoba, Canada, and identify factors associated with mortality among migrants.

**Methods:**

We conducted a retrospective matched-cohort study to examine all-cause and premature mortality of 355,194 international immigrants, interprovincial migrants, and long-term Manitoba residents (118,398 in each group) between January 1985 and March 2019 using linked administrative databases. Poisson regression was used to estimate adjusted incidence rate ratios (aIRR) with 95% confidence intervals (CI).

**Results:**

The all-cause mortality risk of international immigrants (2.3 per 1000 person-years) and interprovincial migrants (4.4 per 1000) was lower than that of long-term Manitobans (5.6 per 1000) (aIRR: 0.43; 95% CI: 0.42, 0.45 and aIRR: 0.81; 95% CI: 0.80, 0.84, respectively). Compared with interprovincial migrants, international immigrants showed lower death risk (aIRR: 0.50; 95% CI: 0.47, 0.52). Similar trends were observed for premature mortality. Among international immigrants, higher mortality risk was observed for refugees, those from North America and Oceania, and those of low educational attainment. Among internal migrants, those from Eastern Canada had lower mortality risk than those migrating from Ontario and Western Canada.

**Conclusion:**

Migrants had a mortality advantage over non-migrants, being stronger for international immigrants than for interprovincial migrants. Among the two migrant groups, there was heterogeneity in the mortality risk according to migrants’ characteristics.

**Supplementary Information:**

The online version contains supplementary material available at 10.17269/s41997-022-00727-4.

## Résumé

**Objectifs:**

Les études sur les écarts dans la mortalité entre les immigrants internationaux et les non-immigrants produisent des résultats mitigés. La mortalité des migrants interprovinciaux est moins étudiée. Nous avons cherché à comparer le risque de mortalité des immigrants internationaux, des migrants interprovinciaux et des résidents à long terme de la province du Manitoba, au Canada, et à cerner les facteurs associés à la mortalité chez les migrants.

**Méthode:**

Nous avons mené une étude de cohorte assortie rétrospective pour examiner la mortalité toutes causes confondues et la mortalité prématurée chez 355 194 immigrants internationaux, migrants interprovinciaux et résidents à long terme du Manitoba (118 398 dans chaque groupe) entre janvier 1985 et mars 2019 à l’aide de bases de données administratives maillées. Par régression de Poisson, nous avons estimé les rapports de taux d’incidence ajustés (RTAa) avec des intervalles de confiance (IC) de 95 %.

**Résultats:**

Le risque de mortalité toutes causes confondues des immigrants internationaux (2,3 pour 1 000 personnes-années) et des migrants interprovinciaux (4,4 pour 1 000) était plus faible que celui des résidents à long terme du Manitoba (5,6 pour 1 000) (RTAa : 0,43; IC de 95 % : 0,42, 0,45 et RTAa : 0,81; IC de 95 % : 0,80, 0,84, respectivement). Comparativement aux migrants interprovinciaux, les immigrants internationaux présentaient un risque de mortalité plus faible (RTAa : 0,50; IC de 95 % : 0,47, 0,52). Des tendances semblables ont été observées pour la mortalité prématurée. Chez les immigrants internationaux, un risque de mortalité plus élevé a été observé chez les réfugiés, les immigrants de l’Amérique du Nord et de l’Océanie et ceux ayant un faible niveau d’instruction. Chez les migrants intérieurs, ceux de l’Est du Canada présentaient un risque de mortalité plus faible que ceux de l’Ontario et de l’Ouest canadien.

**Conclusion:**

Les migrants présentaient un avantage sur le plan de la mortalité par rapport aux non-migrants; cet avantage était plus prononcé chez les immigrants internationaux que chez les migrants interprovinciaux. Dans ces deux groupes de migrants, il y avait hétérogénéité dans le risque de mortalité selon les caractéristiques des migrants.

## Introduction

Studies on the health status of migrants relative to that of non-migrants have produced mixed results. Some studies show immigrant advantage (Abraído-Lanza et al., 1999; Anson, 2004; Rosenwaike & Hempstead, 1990; Sharma et al., 1990) while others show more risk (DesMeules et al., 2005; Gadd et al., 2006; Hollander et al., 2012; Moullan & Jusot, 2014; Norredam et al., 2012; Sundquist & Li, 2006). These heterogeneous results are driven by different study populations, different baseline risk in the host population used for comparisons, different composition of immigrants, and different study methods. For example, in countries that predominantly receive immigrants based on humanitarian grounds (i.e., refugees), such as Sweden, immigrants exhibit higher mortality and heart disease than the native-born (Gadd et al., 2006; Sundquist & Li, 2006). Refugees appear to have a higher mortality risk because of the adverse circumstances preceding or acting during the migration process (DesMeules et al., 2005; Hollander et al., 2012; Norredam et al., 2012). On the other hand, studies conducted in countries that mainly receive economic migrants (i.e., those who self-select themselves for migration), such as Canada and the United States, generally reported lower mortality rates among international immigrants relative to non-migrants (Abraído-Lanza et al., 1999; Anson, 2004; Khan et al., 2017; Rosenwaike & Hempstead, 1990; Sharma et al., 1990), including all-cause mortality and cause-specific mortality (Ng & LHAD Research Team, 2011; Omariba et al., 2014). The concept of migrant mortality advantage (MMA), closely related to the Healthy Immigrant Paradigm (Beiser, 2005), reflects such empirical observations in high-income countries. However, the exact mechanisms behind the low mortality risk among immigrants remain unclear.

Potential mechanisms behind the MMA include selection of healthier individuals for migration, the “salmon bias”, and convergence over time. Selective migration may involve self-migration (i.e., the propensity of healthier individuals to emigrate) and immigration admission policies of the receiving country. Canada selects individuals based on a points system that rewards higher education, work experience, and other attributes conducive to adaptation and success in the labour market. In addition, immigration to Canada may be denied based on a medical examination that makes inadmissible individuals who may pose a danger to public health or safety, or put an excessive demand on health or social services. Selective migration is supported by evidence of favourable sociodemographic characteristics, which are associated with health behaviours and outcomes that are better than those of non-migrants, including mortality (Ali et al., 2004; DesMeules et al., 2005; Omariba et al., 2014). A second popular hypothesis, called the “Salmon Bias”, purports that many migrants return to their homeland after temporary employment, retirement, or severe illness, meaning that their deaths occur in their native land and therefore cannot be captured by the information systems of the country of immigration. The lower mortality of immigrants thus is regarded under this hypothesis as an artefact of including immigrants in the denominator but not in the numerator, creating a “statistically immortal” bias that artificially deflates mortality rates of immigrants (Abraído-Lanza et al., 1999; Wallace & Kulu, 2018). Finally, comparisons of mortality between immigrants and non-immigrants may be affected by a process of convergence (Beiser, 2005) related to duration of residence. While recent immigrants in Canada are generally healthier than the Canadian-born population, exposure to the physical, social, and cultural environment of the destination country may erode the health advantage of recent immigrants (Beiser, 2005; Ng, 2011).

Less is known about the health and mortality of interprovincial migrants. International and interprovincial migration are both influenced by various factors associated with migration (Westphal, 2016; Wingate & Alexander, 2006), such as prospects of upscale income mobility, employment, and social well-being. However, although internal migrants may need to cope with regional adjustments, they do not face challenges specific to foreign migrants such as official language barriers, cultural and political differences, socialization in the local culture, networking, educational gaps, employment and health service access barriers, difficulties of regulations for getting citizenship, and assimilation challenges. Building on the selective migration hypotheses, we expect that both international immigrants and interprovincial migrants will have lower mortality than non-migrants. However, the protective effect of migration is hypothesized to be correlated with the degree of migration, being greater among international immigrants (strong selection) than among interprovincial migrants (weaker selection) for whom the migration experience is less extreme. A secondary objective was to explore mortality differentials among subgroups of international immigrants, defined according to immigrant characteristics such as secondary migration, refugee status, and region of origin, and according to the province of origin of interprovincial migrants.

## Methods

### Data sources

Information on international immigrants was obtained from the Immigration, Refugees and Citizenship Canada (IRCC) Permanent Resident database that contains the landing records of all legal immigrants to Canada from January 1, 1985 to December 31, 2017. This database also contains sociodemographic and immigration characteristics such as refugee status, country of birth, and knowledge of official languages. The Manitoba Health Insurance Registry (the Registry) was used to identify all Manitoba residents who had registered for provincial universal health care coverage between January 1, 1970 and March 31, 2019, some of whom may be international immigrants who settled in other Canadian provinces before 2018 but moved to Manitoba at any time until March 2019. Internal migrants from other Canadian provinces were also identified in the Registry from 1985 onwards. Mortality data were also obtained from the Registry, since death is a key reason for cancellation of health care coverage.

### Study population

We included Manitoba residents of all ages. We included migrants who registered in the Registry between January 1, 1985 and March 31, 2014. The starting point was chosen because of data availability and to allow for a maximum follow-up of 34 years up until March 31, 2019. The exclusion of individuals immigrating after March 31, 2014 was to allow a minimum of 5 years of follow-up until the end of the study period on March 31, 2019. Temporary residents, including students, work permits, and refugee claimants waiting for a decision, could not be included. To estimate the risk of premature mortality, persons aged more than 70 years were censored.

### Study design

We conducted a data linkage retrospective population-based matched-cohort study. The linkage of the IRCC Permanent Resident database with the Registry is of high quality, with a linkage rate of 96% (Urquia et al., 2021). The use of retrospective data made it possible to examine the mortality experience of a large pool of immigrants and non-immigrants over three decades.

For meaningful comparisons of mortality according to the type of migrants, international immigrants were 1:1 matched to long-term Manitobans and interprovincial migrants, as follows. First, international immigrants were hard matched without replacement to long-term Manitobans on birth year, sex, and place of residence at the time of their first registration for health care coverage in Manitoba. Second, due to the smaller pool of interprovincial migrants available for matching, international immigrants were matched without replacement to interprovincial migrants based on a propensity score with a caliper of 0.01. The propensity score was based on a logistic model regressing the probability of being an international immigrant and an interprovincial migrant, based on age, sex, place of first residence in Manitoba, and date of immigration to Manitoba (within 5 years). Because the same international immigrant was matched to one long-term Manitoban and one interprovincial migrant, the three formed a matching triad. The adequacy of the matching was deemed satisfactory based on comparisons of sociodemographics, with standardized differences consistently below 10%.

To explore mortality risk within international and interprovincial migrants according to the immigration characteristics and the province of origin, respectively, no matching was needed, and all individuals within each group were included because comparisons were internal to immigrants or interprovincial migrants, respectively.

### Variable definitions

#### Outcome variables

We studied all-cause mortality and premature mortality (deaths up until age 70). The outcome variable was death between January 1985 and March 2019.

#### Comparison groups

*International immigrants* were all Manitoba residents with a landing record in the IRCC’s Permanent Resident database. International migrants were further subclassified as refugees, not refugees, primary immigrants (migrated from the country of origin), and secondary immigrants (migrated from a country other than their country of birth) (Urquia et al., 2010; Wanigaratne et al., 2016).

*Interprovincial migrants* were those who migrated to Manitoba from other Canadian provinces and were not foreign-born. Long-term Manitobans, the reference group, include those who registered to the provincial public health care insurance since birth due to being born in Manitoba after 1970 and came under insurance coverage since birth and those who resided in Manitoba before the creation of the Registry in 1970.

#### Sociodemographic characteristics

Sociodemographic characteristics available for the whole population were only obtained from the Registry and included year of first registration for health coverage, age, sex, relationship status, and place of residence. Place of residence was used to derive rural residence and neighbourhood income quintiles based on Canadian censuses at the dissemination area level, the smallest geographic unit at which census data are aggregated. The Registry also included the province from which internal migrants originated. Variables only available for international immigrants were obtained from the IRCC Permanent Resident database, including marital status at arrival, educational attainment, refugee status, country of birth, and last permanent residence. The two groups of migrants were categorized by decade of arrival and age at the start of follow-up, which extended to Manitobans who were matched to them.

#### Data analysis

Kaplan-Meier curves were used to describe the mortality experience of the three groups. Survival time was defined as time from immigration to Manitoba to death, or censorship due to move out-of-province (i.e., date of cancellation of health care coverage) or end of study period, expressed as person-years. For long-term Manitoba residents, we used the date of arrival of their matched immigrants as the date of start of the follow-up. Poisson regression models assessed the association between migrant status and all-cause and premature mortality, expressed as incidence rate ratios that account for differential follow-up time by using the log of person-years of follow-up as an offset variable. For the main analysis comparing the mortality risk of international and interprovincial migrants with that of non-migrants, we used conditional Poisson regression, which takes into account the matching between the comparison groups, according to age, sex, and region of residence at the time of arrival in Manitoba. Models were further adjusted for year of start of health care coverage, relationship status, neighbourhood income quintile, and rural residence. In analyses restricted to international and interprovincial migrants, respectively, where the focus was to explore heterogeneity of risk according to various immigration characteristics within each of these two groups, no matching was needed because the comparisons were internal to immigrants and unconditional Poisson regression analysis was used instead. All analyses were performed in SAS 9.4 (SAS Institute, Cary, NC, USA).

#### Ethics approval

Use of the data was approved by the University of Manitoba Health Research Ethics Board (HS22881 (H2019:216)) and was reviewed for data privacy and approved by the Government of Manitoba’s Health Information and Privacy Committee (File No. 2019/2020-17).

## Results

### Description of the final sample sizes

There were 263,711 international immigrants. Of these, 55,745 were excluded if they arrived on or after April 1, 2014, to allow for a minimum of 5 years of follow-up. We also excluded 552 immigrants with invalid postal code information. The final sample size for this group was 207,414.

There were 278,289 interprovincial migrants whose health care coverage started from 1985 to 2017. After exclusion of 24,661 individuals who did not have an international immigrant match arriving after April 2014 and 1336 with invalid postal code information, the final sample size for interprovincial migrants was 252,292.

In the Registry, there were 727,341 long-term Manitoba residents whose coverage started before 1970 and 692,515 born in the province since then, adding to 1,419,856 Manitobans. After matching on birth year, sex, and place of residence, the ratio of immigrants and interprovincial migrants to long-term Manitobans was 1. The final sample sizes for the matched analyses included 355,194 individuals (118,398 in each comparison group).

### Characteristics of the matched study population

Table 1 depicts the characteristics of the three groups. There was an increasing gradient in crude mortality rates from international to interprovincial to long-term Manitobans. There were virtually no differences between the groups with respect to the matching variables year of start of the follow-up, age groups and sex. Differences between groups in the distribution of income quintiles and rural residence were greatly reduced because of the matching by place of residence. International immigrants were the most likely to have spouses, followed by interprovincial migrants. Most immigrants immigrated from Asia, followed by Europe. Most interprovincial migrants to Manitoba migrated from Ontario and Alberta, followed by Saskatchewan and British Columbia.

**Table 1:** Characteristics of the matched study population

	International immigrants (*N*=118,398)	Interprovincial migrants (*N*=118,398)	Long-term Manitobans (*N*=118,398)	International immigrants	Interprovincial migrants	Long-term Manitobans
	Person-years (number of deaths)	Person-years (number of deaths)	Person-years (number of deaths)	Rate (per 1000 person-years)/column percent	Rate (per 1000 person-years)/column percent	Rate (per 1000 person-years)/column percent
All-cause mortality	1,385,665 (3184)	915,299 (4011)	1,654,873 (9215)	2.30	4.38	5.57
Premature mortality	1,326,823 (1363)	882,754 (1905)	1,581,687 (5396)	1.03	2.16	3.41
Year of start of follow-up						
1985–1994	627,737	388,328	762,328	45.30	43.61	46.07
1995–2004	432,000	281,165	524,574	31.18	31.58	31.70
2005–2014	325,929	245,806	367,971	23.52	246.86	22.24
Age at start of follow-up						
0–14	377,817	265,320	422,284	27.27	28.99	25.52
15–24	271,809	144,436	288,331	19.62	15.78	17.42
25–44	549,157	380,806	709,895	39.63	41.60	42.90
45–64	150,007	100,970	193,731	10.83	11.03	11.71
65–84	36,302	21,507	40,152	2.62	2.35	2.43
85 and above	573	2260	479	0.04	0.25	0.03
Male	695,173	454,600	822,291	50.17	49.67	49.69
Female	690,492	460,699	832,582	49.83	50.33	50.31
Neighbourhood income						
Quintile 1	466,252	222,603	555,204	33.65	24.32	33.55
Quintile 2	287,532	179,553	296,698	20.75	19.62	17.93
Quintile 3	233,384	165,801	304,934	16.84	18.11	18.43
Quintile 4	219,272	163,660	246,540	15.82	17.88	14.90
Quintile 5	172,054	178,489	206,503	12.42	19.50	12.48
Quintile N/A	7171	5193	44,993	0.52	0.57	2.72
Rural residence	229,342	176,725	298,610	16.55	19.31	18.04
Urban residence	1,156,323	738,574	1,356,263	83.45	80.69	81.96
Relationship status: has spouse	518,125	277,268	456,282	37.39	30.29	27.57
Relationship status: has no spouse	867,539	638,031	1,198,591	62.61	69.71	72.43
Immigrant characteristics						
Refugee	260,882			18.83		
Not refugee	1,124,783			81.17		
Secondary migration	154,426			11.14		
Primary migration	1,231,238			88.86		
Marital status						
Single never married	727,764			52.52		
Married or common law	608,711			43.93		
Divorced/separated/widowed	37,189			2.68		
Unknown	12,000			0.87		
Educational attainment						
Secondary or less	677,552			48.90		
Some post-secondary	244,250			17.63		
University degree (reference)	237,544			17.14		
Unknown	226,319			16.33		
Knowledge of English and/or French	718,495			51.85		
No knowledge of English and/or French	667,170			48.15		
World region of origin						
Southeast Asia	403,278			29.10		
South Asia	132,031			9.53		
Rest of Asia	177,953			12.84		
East Europe	145,083			10.47		
Western Europe (reference)	204,324			14.75		
North Africa	20,119			1.45		
East Africa	52,045			3.76		
Rest of Africa	42,191			3.04		
North America and Oceania	47,644			3.44		
Latin America and Caribbean	160,594			11.59		
Unknown region of birth	403			0.03		
Province of origin						
Manitoba			1,654,873			100.00
British Columbia		140,919			15.40	
Alberta		203,271			22.21	
Saskatchewan		149,465			16.33	
Ontario (reference)		320,921			35.06	
Quebec		35,567			3.89	
Atlantic Canada (NB, NS, PE)		53,387			5.83	
Arctic (NT, YK, NU)		11,769			1.29	

Abbreviations: *NB*, New Brunswick; *NS*, Nova Scotia; *PE*, Prince Edward Island; *NT*, Northwest Territories; *YK*, Yukon; *NU*, Nunavut

### All-cause mortality and premature mortality rates

**Fig. 1:**
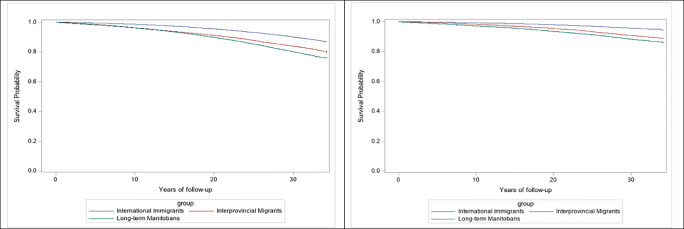
Survival curves for all-cause (left) and premature mortality (right) among matched international immigrants, interprovincial migrants, and long-term Manitobans

The all-cause mortality and premature mortality rates in the matched sample were highest for Manitobans (5.57 and 3.41 per 1000 person-years, respectively), followed by interprovincial migrants (4.38 and 2.16, respectively) and then international immigrants (2.30 and 1.03, respectively) (Table 1). Compared to long-term Manitobans, the adjusted risk of death of international migrants was substantially lower than that of Manitobans (aIRR: 0.43; 95% CI: 0.42, 0.45 for all-cause mortality and aIRR: 0.35; 95% CI 0.33–0.37 for premature mortality) (Table 2). Similarly, interprovincial migrants were less likely to experience all-cause and premature mortality relative to Manitobans (aIRR: 0.81; 95% CI: 0.80, 0.84 and aIRR: 0.70; 95% of CI: 0.66, 0.74, respectively). For all-cause and premature mortality, the rates of international migrants were significantly lower compared to those of interprovincial migrants (aIRR: 0.54; 95% CI: 0.51, 0.56 and 0.49, 95% CI 0.46, 0.53, respectively). These associations did not appreciably differ according to sex (Table 2). In analyses stratified by age at arrival groups, the mortality advantage of international immigrants was observed in all of them (data not shown), as it was observed in analyses stratified by period of the start of the follow-up (1985–1994, 1995–2004 and 2005–2014) (Supplemental Tables S1 and S2). Kaplan-Meier curves (Fig. 1) show that the mortality advantage of international immigrants was apparent across time after migration. The downward curvilinear pattern for all three groups, more visible for all-cause mortality (Fig. 1, left), indicates an acceleration of the risk of death, parallel to increasing age and time since migration.

**Table 2:** All-cause and premature mortality among matched international migrants (*N*=118,398), interprovincial migrants (*N*=118,398), and long-term Manitobans (*N*=118,398), overall and by sex

	All-cause mortality		Premature mortality	
	IRR (95% CI)	AIRR^a^ (95% CI)	IRR (95% CI)	AIRR^a^ (95% CI)
All				
Immigrants vs. long-term Manitobans (ref)	0.40 (0.38, 0.41)	0.43 (0.42, 0.45)	0.30 (0.28, 0.32)	0.35 (0.33, 0.37)
Interprovincial migrants vs. long-term Manitobans (ref)	0.77 (0.74, 0.80)	0.81 (0.80, 0.84)	0.63 (0.61, 0.66)	0.70 (0.66, 0.74)
Immigrants vs. interprovincial migrants (ref)	0.52 (0.49, 0.54)	0.54 (0.51, 0.56)	0.47 (0.44, 0.51)	0.49 (0.46, 0.53)
Female				
Immigrants vs. long-term Manitobans (ref)	0.41 (0.39, 0.44)	0.41 (0.39, 0.44)	0.28 (0.25, 0.31)	0.28 (0.26, 0.31)
Interprovincial migrants vs. long-term Manitobans (ref)	0.79 (0.74, 0.83)	0.86 (0.81, 0.91)	0.59 (0.54, 0.64)	0.65 (0.60, 0.70)
Immigrants vs. interprovincial migrants (ref)	0.53 (0.49, 0.55)	0.54 (0.51, 0.58)	0.47 (0.42, 0.53)	0.43 (0.38, 0.48)
Male				
Immigrants vs. long-term Manitobans (ref)	0.37 (0.35, 0.39)	0.41 (0.39, 0.43)	0.31 (0.29, 0.33)	0.36 (0.33, 0.39)
Interprovincial migrants vs. long-term Manitobans (ref)	0.77 (0.73, 0.81)	0.80 (0.76, 0.83)	0.66 (0.62, 0.70)	0.70 (0.66, 0.75)
Immigrants vs. interprovincial migrants (ref)	0.48 (0.45, 0.52)	0.52 (0.49, 0.55)	0.47 (0.43, 0.51)	0.52 (0.47, 0.56)

Abbreviations: *IRR*, incidence rate ratio; *AIRR*, adjusted incidence rate ratio; *CI*, confidence intervals

1:1 matching based on sex, age at the start of the follow-up, and place of residence at the start of the follow-up

Estimates obtained with conditional Poisson regression with person-years as an offset variable

^a^Adjusted for relationship status, neighbourhood income quintile, and rural residence

### Mortality according to the characteristics of foreign-born migrants

Table 3 presents the rates for all-cause and premature death among all (matched and non-matched) international immigrants. Refugees had higher mortality risk relative to non-refugees, after adjustment. Secondary immigrants had higher mortality rates before but not after adjustment, due to being older on average than primary immigrants. Single never married individuals had higher mortality risk than those with a spouse, after adjustment. An inverse relationship existed between education level and risk of death; immigrants who had secondary education or less and those with some post-secondary education had higher all-cause and premature mortality risk than those with a university degree. Southeast Asians, South Asians, the rest of Asians, and those from Latin America and the Caribbean had a lower risk of all-cause mortality than Western Europeans, but these advantages were not observed for premature mortality. Immigrants from North America and Oceania, a group composed of 86.8% of immigrants from the USA, had a higher risk of both all-cause and premature mortality. A positive association among Eastern Europeans was observed for premature mortality but was no longer statistically significant for all-cause mortality, after adjustment.

**Table 3:** Crude and adjusted incidence rate ratios for all-cause and premature mortality among international immigrants (*N*=207,414)

	All-cause mortality					Premature mortality				
	No. of deaths	Person-years	Rate (× 1000 p-y)	IRR (95% CI)	AIRR^a^ (95% CI)	No. of deaths	Person-years	Rate (× 1000 p-y)	IRR (95% CI)	AIRR^a^ (95% CI)
Immigrant characteristics										
Refugee										
Yes	490	339,028	1.45	**0.78 (0.71 0.86)**	**1.27 (1.13 1.42)**	383	335,328	1.14	**1.35 (1.20 1.51)**	**1.38 (1.19 1.59)**
No	3336	1,797,935	1.86	1.00 (ref)	1.00 (ref)	1471	1,735,167	0.85	1.00 (ref)	1.00 (ref)
Secondary immigrant										
Yes	575	249,598	2.30	**1.34 (1.22 1.46)**	1.06 (0.97 1.17)	232	239,907	0.97	1.09 (0.95 1.25)	0.99 (0.85 1.15)
No	3251	1,887,365	1.72	1.00 (ref)	1.00 (ref)	1622	1,830,588	0.89	1.00 (ref)	1.00 (ref)
Marital status										
Single never married	545	1,076,073	0.51	**0.21 (0.19 0.23)**	**1.27 (1.13 1.43)**	474	1,074,019	0.44	**0.34 (0.30 0.37)**	**1.23 (1.07 1.41)**
Married or common law	2375	996,344	2.38	1.00 (ref)	1.00 (ref)	1256	954,608	1.32	1.00 (ref)	1.00 (ref)
Divorced, separated, or widowed	820	51,124	16.04	**6.73 (6.22 7.28)**	1.08 (0.98 1.19)	92	30,421	3.02	**2.30 (1.86 2.84)**	0.96 (0.77 1.20)
Unknown	86	13,422	6.41	**2.69 (2.17 3.33)**	1.02 (0.82 1.27)	32	11,447	2.80	**2.12 (1.50 3.02)**	1.06 (0.75 1.52)
Educational attainment										
Secondary or less	2233	949,793	2.35	**2.08 (1.89 2.29)**	**1.41 (1.27 1.58)**	925	907,527	1.02	**1.30 (1.15 1.47)**	**1.48 (1.29 1.70)**
Trade/certificate/post-secondary	641	377,126	1.70	**1.50 (1.34 1.69)**	**1.28 (1.14 1.44)**	440	370,466	1.19	**1.51 (1.32 1.74)**	**1.35 (1.17 1.57)**
University degree	515	455,966	1.13	1.00 (ref)	1.00 (ref)	353	449,517	0.79	1.00 (ref)	1.00 (ref)
Unknown	437	354,078	1.23	1.09 (0.96 1.24**)**	**1.26 (1.09 1.47)**	136	342,985	0.40	**0.51 (0.41 0.62)**	1.25 (0.99 1.58)
Knowledge of English and/or French										
Yes	1980	1,177,018	1.68	0.87 (0.82 0.93)	1.03 (0.96 1.12)	1024	1,148,851	0.89	0.99 (0.90 1.09)	0.96 (0.85 1.07)
No	1846	959,945	1.92	1.00 (ref)	1.00 (ref)	830	921,644	0.90	1.00 (ref)	1.00 (ref)
World region of origin										
Southeast Asia	1359	724,638	1.88	0.94 (0.85 1.04)	**0.88 (0.78 0.98)**	633	698,231	0.91	1.11 (0.95 1.30)	1.14 (0.96 1.35)
South Asia	403	248,152	1.62	0.81(0.71 0.93)	**0.74 (0.64 0.85)**	199	238,343	0.84	1.02 (0.84 1.24)	1.03 (0.84 1.28)
Rest of Asia	410	277,215	1.48	0.74 (0.65 0.84)	**0.67 (0.58 0.77)**	204	268,360	0.76	0.93 (0.77 1.13)	0.86 (0.70 1.06)
East Europe	453	193,431	2.34	**1.17 (1.03 1.33)**	1.11 (0.97 1.27)	246	187,564	1.31	**1.60 (1.33 1.94)**	**1.28 (1.05 1.56)**
Western Europe	502	251,572	2.00	1.00 (ref)	1.00 (ref)	200	244,997	0.82	1.00 (ref)	1.00 (ref)
North Africa	34	33,858	1.00	**0.50 (0.35 0.71)**	0.94 (0.66 1.34)	24	33,538	0.72	0.88 (0.57 1.34)	1.16 (0.75 1.79)
East Africa	89	89,140	1.00	**0.50 (0.40 0.63)**	0.89 (0.70 1.13)	77	88,623	0.87	1.06 (0.82 1.38)	1.13 (0.85 1.50)
Rest of Africa	70	69,503	1.01	**0.50 (0.39 0.65)**	0.85 (0.65 1.09)	49	68,694	0.71	0.87 (0.64 1.19)	1.11 (0.80 1.53)
North America and Oceania	203	55,995	3.63	**1.82 (1.54 2.14)**	**1.27 (1.07 1.50)**	72	53,803	1.34	**1.64 (1.25 2.15)**	**1.57 (1.19 2.06)**
Latin America and Caribbean	302	192,949	1.57	**0.78 (0.68 0.90)**	**0.77 (0.67 0.89)**	150	187,901	0.80	0.98 (0.79 1.21)	0.82 (0.66 1.01)

Abbreviations: *IRR*, incidence rate ratio; *AIRR*, adjusted incidence rate ratio; *CI*, confidence intervals

Estimates obtained with unconditional Poisson regression with person-years as an offset variable

^a^Adjusted for year of start of the follow-up, age at the start of the follow-up (cubic term = age age*age age*age*age), sex, neighbourhood income quintile, rural residence, and all the other variables in the table

### Mortality among interprovincial migrants

Table 4 shows that migrants from all provinces except British Columbia—particularly those from Eastern Canada—had lower death risk than those from Ontario, the most populous Canadian province.

**Table 4:** Crude and adjusted incidence rate ratios for all-cause and premature mortality among interprovincial migrants (*N *= 252,292)

	All-cause mortality					Premature mortality				
	No. of deaths	Person-years	Rate (per 1000 person-years)	IRR (95% CI)	AIRR^a^ (95% CI)	No. of deaths	Person-years	Rate (per 1000 person-years)	IRR (95% CI)	AIRR^a^ (95% CI)
Province of origin										
British Columbia	2558	337,141	7.59	**1.41 (1.34 1.48)**	1.01 (0.96 1.06)	799	313,522	2.55	**1.18 (1.08 1.28)**	1.05 (0.97 1.15)
Alberta	2113	519,663	4.07	**0.75 (0.72 0.79)**	1.03 (0.97 1.08)	1115	502,257	2.22	1.03 (0.95 1.11)	1.05 (0.97 1.14)
Saskatchewan	2264	456,689	4.96	**0.92 (0.87 0.97)**	1.00 (0.94 1.05)	803	436,057	1.84	**0.85 (0.78 0.93)**	0.94 (0.86 1.02)
Ontario	3939	730,385	5.39	1.00 (ref)	1.00 (ref)	1501	693,123	2.17	1.00 (ref)	1.00 (ref)
Quebec	446	86,044	5.18	0.96 (0.87 1.06)	**0.85 (0.77 0.94)**	152	80,982	1.88	0.87 (0.73 1.02)	**0.78 (0.66 0.93)**
Atlantic Canada (NB, NS, PE)	433	159,642	2.71	**0.50 (0.46 0.56)**	**0.82 (0.74 0.90)**	218	155,805	1.40	**0.65 (0.56 0.74)**	**0.77 (0.66 0.88)**
Arctic (NT, YK, NU)	122	35,692	3.42	**0.63 (0.53 0.76)**	0.97 (0.81 1.16)	76	34,585	2.20	1.02 (0.81 1.28)	0.97 (0.77 1.22)

Abbreviations: *IRR*, incidence rate ratio; *AIRR*, adjusted incidence rate ratio; *CI*, confidence intervals; *NB*, New Brunswick; *NS*, Nova Scotia; *PE*, Prince Edward Island; *NT*, Northwest Territories; *YK*, Yukon; *NU*, Nunavut

Estimates obtained with unconditional Poisson regression with person-years as an offset variable

^a^Adjusted for start of follow-up year, age at start of follow-up (cubic term = age age*age age*age*age), sex, relationship status, neighbourhood income quintile, and rural or urban residence

## Discussion

### Main findings

We found that both international immigrants and interprovincial migrants had lower mortality risk than the local Manitoban population. The migrant mortality advantage, however, was stronger for international than for interprovincial migrants. Among the two migrant groups, there was heterogeneity in the mortality risk according to migrants’ characteristics, mainly higher risk among refugees, those from North America and those with low educational attainment. Internal migrants from the eastern Canadian provinces had lower mortality risk.

### Limitations

There were some limitations to this study. Sociodemographic information was only collected from the permanent residence data and the Registry. Temporary residents, such as international visa students, work permit holders, and refugee claimants waiting for a decision, were not included in this study. Although immigrants lost to follow-up were censored, it is possible that some of them may have died shortly after remigrating and could not contribute to the numerator, thus deflating their mortality risk (Andersson & Drefahl, 2017; Wallace & Kulu, 2018). This study could not distinguish those who left the province and returned to their country or province of origin due to ill health from those who remigrated to another destination and may be healthier. Some immigrant characteristics were measured at the time of arrival and could not be updated at a later time, such as education and marital status. This may have affected the efficiency of the adjustment and contributed to residual confounding. We also lacked measures of morbidity and information on the medical history of migrants before arriving in the country and the province. Finally, since the focus of the study was to examine the overall mortality gradient of international and interprovincial migrants and non-migrants, more detailed examinations of specific mechanisms that may affect particular subgroups defined by place of origin, period of arrival, and other migrant characteristics were beyond the scope of this study.

### Interpretation

This study confirmed the lower mortality risk among immigrants in Canada. We found that both international and interprovincial migrants have a mortality advantage over the local Manitoban population. We also found that interprovincial migrants had death rates that are intermediate between those of the other two groups. This suggests that while migrants in general are healthier than non-migrants, international migration is associated with a stronger protective effect than internal migration, which may be a reflection of a stronger selection for migration. While interprovincial migrants share with the local population the language, political and cultural similarities, their migration decisions may be driven by better health status, which may be behind their mortality advantage (Westphal, 2016). However, since the migration experience of interprovincial migrants is less radical than that of international immigrants, so is the effect. Results also indicate that the difference between the rates of all-cause and premature mortality was higher between the two groups of migrants over Manitobans than between the two migrant groups. This observation suggests an influence of a lower life expectancy of Manitobans in general and of Indigenous groups in particular, which are prevalent in Manitoba and known to have lower life expectancy than the rest of the Manitoba population (Katz et al., 2021).

In analyses restricted to international immigrants, we found that refugees had higher all-cause and premature mortality risk than non-refugees, after adjustment. This association may reflect specific determinants of health not shared by their non-refugee counterparts, such as involuntary and forced migration, political unrest and persecution, possible exposure to refugee camps, and limited access to quality health care and living conditions during the migration process (Wanigaratne et al., 2016). In addition, in 2002, the Canadian Immigration and Refugee Protection Act updated immigration selection criteria by exempting certain immigrant classes (e.g., refugees and family class) from inadmissibility on health grounds (Government of Canada, 2001), which may have also contributed to the admission of less healthy refugees. Secondary immigrants exhibited higher unadjusted mortality rates, due to being older on average than primary immigrants, but the associations disappeared after adjustment. These findings do not support a secondary migration advantage in mortality that has been observed in reproductive health (Urquia et al., 2010), particularly among non-refugees (Wanigaratne et al., 2016). Finally, there were variations according to immigrants’ birthplace. Consistent with previous Canadian studies (Ng, 2011; Ng & LHAD Research Team, 2011), Asian immigrants had low mortality rates, presumably due to more favourable diet and health behaviours. East Europeans exhibited higher premature mortality in our study, which may be related to a higher prevalence of risk factors for chronic diseases and death, such as smoking, alcohol, and poor diet (McKee & Shkolnikov, 2001; Powles et al., 2005). Less clear are the reasons behind the excess mortality among those from North America and Oceania, a subgroup composed predominantly of immigrants from the USA. The possibility that migration from the USA to Manitoba is associated with a negative health selection should be investigated in future studies, without disregarding the potential contribution of various First Nations or American Indian Nations, which have cross-border rights due to their original territories and family and social life being disrupted by the US-Canada border (Assembly of First Nations, 2020).

In analyses restricted to interprovincial migrants, we found that migrants from the province of British Columbia had higher unadjusted mortality risk than migrants from Ontario. However, the excess risk was explained by observed individual characteristics. More robust is the lower mortality observed among those from Eastern Canada (Atlantic Canada and Quebec). Although we lacked additional data (i.e., socioeconomic status before migration, occupation, or reasons for migration) that may help explain these patterns, the selection hypothesis may suggest that migration from better-off places like British Columbia, which has a strong economy and the highest life expectancy among all Canadian provinces (Zhang & Rasali, 2015), may be negatively selected for health, whereas migration from provinces with economies either weaker than or similar to Manitoba’s, such as those of Eastern Canada (Statistics Canada, 2021), may be positively selected for health.

## Conclusion

This study found a mortality gradient according to the degree of migration, being lower among international immigrants, intermediate among interprovincial migrants, and highest among long-term Manitobans. This gradient is likely to reflect different levels of selection for migration; the stronger the selection, the lower the mortality. Our findings suggest that efforts to achieve gains in life expectancy in the province of Manitoba may be better focused on the local population. Despite an overall mortality advantage, heterogeneity in mortality risk exists within immigrants. Understanding the protective factors that underlie the migrant mortality advantage may help with devising prevention strategies for the local population and the migrant subgroups at higher risk, such as refugees and those from the USA.

## Contributions to knowledge

What does this study add to existing knowledge?

- Our study provides novel evidence of the role of selective migration on mortality by including interprovincial migrants, a group rarely studied.
- The stronger the selection associated with migration, the stronger the mortality advantage, as evidenced by the increasing mortality gradient from international immigrants to interprovincial migrants to long-term Manitoba residents.
- The study also identifies factors associated with mortality differentials within migrant groups.

What are the key implications for public health interventions, practice, or policy?

- Given the mortality advantage of both international immigrants and interprovincial migrants with respect to Manitoba residents, efforts to achieve gains in life expectancy in the province of Manitoba may be better focused on the local population and on high-risk migrant groups, such as refugees.

## Supplementary Information

ESM 1 (DOCX 26 kb)

## Data Availability

This study’s data were accessed from the Manitoba Population Research Data Repository at Manitoba Centre of Health Policy. Use of the provincial data was approved by Manitoba Health, Seniors and Active Living. The study data can be accessed upon obtaining the required permissions.

## Code availability

All programming was performed with SAS. SAS code is available from the authors upon request.
