# Supplementary material for: Assessing the migrant mortality advantage among foreign-born and interprovincial migrants in Manitoba, Canada
Source: Can J Public Health. 2022 Dec 27;114(3):441–52. doi: 10.17269/s41997-022-00727-4 (PMC10156882; doi:10.17269/s41997-022-00727-4)
Supplement: Supplementary file 1 — (DOCX 26 kb) [file 41997_2022_727_MOESM1_ESM.docx]

**Supplementary Table 1: Crude mortality rates by sex and start of follow-up among matched International Migrants (N=118,398), Interprovincial Migrants (N=118,398), and Long-Term Manitobans (N=118,398), overall, by sex and by start of follow-up**

|  | **All-cause mortality** | | | **Premature mortality** | | |
| --- | --- | --- | --- | --- | --- | --- |
|  | **No of deaths** | **Person-years** | **Rate (per 1000 person-years)** | **No of deaths** | **Person-years** | **Rate (per 1000 person-years)** |
| **International immigrants (N=118,398)** |  |  |  |  |  |  |
| **All** | 3184 | 1,385,665 | 2.30 | 1363 | 1,326,823 | 1.03 |
| **By sex** |  |  |  |  |  |  |
| Male | 1632 | 695,173 | 2.35 | 854 | 672,292 | 1.27 |
| Female | 1552 | 690,492 | 2.25 | 509 | 654,531 | 0.78 |
| **By start of follow-up** |  |  |  |  |  |  |
| 1985-1994 | 2180 | 627,737 | 3.47 | 850 | 587,458 | 1.45 |
| 1995-2004 | 720 | 432,000 | 1.67 | 342 | 418,890 | 0.82 |
| 2005-2014 | 284 | 325,929 | 0.87 | 171 | 320,476 | 0.53 |
| **Interprovincial migrants (N=118,398)** |  |  |  |  |  |  |
| **All** | 4011 | 915,299 | 4.38 | 1905 | 882,754 | 2.16 |
| **By sex** |  |  |  |  |  |  |
| Male | 2124 | 454,600 | 4.67 | 1190 | 441,425 | 2.70 |
| Female | 1887 | 460,699 | 4.10 | 715 | 441,329 | 1.62 |
| **By start of follow-up** |  |  |  |  |  |  |
| 1985-1994 | 1793 | 388,328 | 4.62 | 989 | 374,360 | 2.64 |
| 1995-2004 | 1219 | 281,165 | 4.34 | 574 | 271,907 | 2.11 |
| 2005-2014 | 999 | 245,806 | 4.06 | 342 | 236,487 | 1.45 |
| **Long-term Manitobans (N=118,398)** |  |  |  |  |  |  |
| **All** | 9215 | 1,654,873 | 5.57 | 5396 | 1,581,687 | 3.41 |
| **By sex** |  |  |  |  |  |  |
| Male | 4921 | 822,291 | 5.99 | 3241 | 795,325 | 4.08 |
| Female | 4294 | 832,582 | 5.16 | 2155 | 786,363 | 2.74 |
| **By start of follow-up** |  |  |  |  |  |  |
| 1985-1994 | 5908 | 762,328 | 7.75 | 3039 | 711,707 | 4.27 |
| 1995-2004 | 2383 | 524,574 | 4.54 | 1640 | 508,012 | 3.23 |
| 2005-2014 | 924 | 367,971 | 2.51 | 717 | 361,968 | 1.98 |

1:1 matching based on sex, age at start of follow-up and place of residence at start of follow-up.

**Supplementary Table 2: All-cause and premature mortality among matched International Migrants (N=118,398), Interprovincial Migrants (N=118,398), and Long-Term Manitobans (N=118,398), by period of start of follow-up**

| **Start of follow-up** | **All-Cause Mortality** | | **Premature Mortality** | |
| --- | --- | --- | --- | --- |
|  | **IRR (95% CI)** | **AIRR ^a^ (95% CI)** | **IRR (95% CI)** | **AIRR ^a^ (95% CI)** |
| **1985-1994** |  |  |  |  |
| Immigrants vs. Long-term Manitobans | 0.42 (0.40, 0.44) | 0.45 (0.43, 0.47) | 0.33 (0.31, 0.36) | 0.37 (0.34, 0.40) |
| Interprovincial Migrants vs. Long-term Manitobans | 0.58 (0.55, 0.61) | 0.76 (0.73, 0.80) | 0.62 (0.57, 0.66) | 0.69 (0.64, 0.74) |
| Immigrants vs. Interprovincial Migrants | 0.73 (0.68, 0.78) | 0.60 (0.56, 0.62) | 0.54 (0.49, 0.59) | 0.54 (0.49, 0.59) |
| **1995-2004** |  |  |  |  |
| Immigrants vs. Long-term Manitobans | 0.35 (0.32, 0.38) | 0.40 (0.37, 0.44) | 0.25 (0.22, 0.28) | 0.30 (0.27, 0.34) |
| Interprovincial Migrants vs. Long-term Manitobans | 0.95 (0.89, 1.02) | 0.77 (0.72, 0.82) | 0.65 (0.59, 0.72) | 0.69 (0.63, 0.76) |
| Immigrants vs. Interprovincial Migrants | 0.36 (0.33, 0.40) | 0.52 (0.48, 0.58) | 0.38 (0.34, 0.44) | 0.44 (0.39, 0.51) |
| **2005-2014** |  |  |  |  |
| Immigrants vs. Long-term Manitobans | 0.33 (0.29, 0.38) | 0.39 (0.34, 0.44) | 0.26 (0.22, 0.31) | 0.33 (0.28, 0.39) |
| Interprovincial Migrants vs. Long-term Manitobans | 1.62 (1.47, 1.77) | 0.83 (0.76, 0.91) | 0.72 (0.64, 0.83) | 0.68 (0.59, 0.77) |
| Immigrants vs. Interprovincial Migrants | 0.21 (0.18, 0.24) | 0.46 (0.41, 0.53) | 0.36 (0.30, 0.44) | 0.49 (0.40, 0.59) |

Abbreviations: IRR: Incidence Rate Ratio; AIRR: Adjusted Incidence Rate Ratio; CI: Confidence Intervals

1:1 matching based on sex, age at start of follow-up and place of residence at start of follow-up.

Estimates obtained with Conditional Poisson regression with person-years as an offset variable.

^a^ Adjusted for relationship status, neighbourhood income quintile and rural residence
